# Dynamics and Crosstalk between Gut Microbiota, Metabolome, and Fecal Calprotectin in Very Preterm Infants: Insights into Feeding Intolerance

**DOI:** 10.3390/nu15224849

**Published:** 2023-11-20

**Authors:** Luyang Hong, Yihuang Huang, Junyan Han, Shujuan Li, Lan Zhang, Siyuan Jiang, Qi Zhou, Xincheng Cao, Weiyin Yu, Yi Yang, Shangyu Hong, Yufeng Zhou, Weili Yan, Yun Cao

**Affiliations:** 1 Department of Neonatology, Children’s Hospital of Fudan University, National Children’s Medical Center, Shanghai 201102, China; 2 NHC Key Laboratory of Neonatal Diseases, Fudan University, Shanghai 201102, China; yyang@shmu.edu.cn (Y.Y.);; 3 State Key Laboratory of Genetic Engineering, School of Life Sciences, Fudan University, Shanghai 200438, China; shangyu_hong@fudan.edu.cn; 4 Department of Clinical Epidemiology, Children’s Hospital of Fudan University, National Children’s Medical Center, Shanghai 201102, China

**Keywords:** preterm infants, feeding intolerance, microbiome, calprotectin, polyamine metabolites, pantothenic acid

## Abstract

Background: Feeding intolerance (FI) is a significant concern in the care of preterm infants, impacting their growth and development. We previously reported that FI is linked to lower fecal calprotectin (FC) levels. This study aims to explore the postnatal dynamics and interplay between microbiota, metabolic profiles, and host immunity in preterm infants with and without FI. Methods: Infants with gestational age <32 weeks or birth weight <1500 g were enrolled at the Children’s Hospital of Fudan University between January 2018 and October 2020. Weekly fecal samples were analyzed for bacterial profiling, metabolome, and calprotectin levels, exploring their longitudinal development and interrelationships. Results: Of the 118 very preterm infants studied, 48 showed FI. These infants experienced an interrupted microbial–immune trajectory, particularly at 3–4 weeks of age, marked by a reduced bacterial abundance, alpha diversity, and FC levels. Metabolic changes in FI were pronounced between 3 and 6 weeks. Pantothenic acid and two polyamine metabolites were closely associated with bacterial abundance and FC levels and negatively correlated with the duration to attain full enteral feeding. Conclusions: FI infants demonstrated compromised microbiome–immune interactions, potentially influenced by specific metabolites. This research underscored the importance of early microbial and metabolic development in the pathogenesis of FI in very preterm infants.

## 1. Introduction

The gastrointestinal system of preterm infants is characterized by immaturity, including underdeveloped gastrointestinal (GI) motility, a fragile mucosal barrier, immature immune function, and inadequate neuroendocrine crosstalk [1]. As a result, feeding intolerance (FI) emerges as a common challenge in the neonatal intensive care unit (NICU). FI in preterm infants is often manifested by abdominal distention, vomiting, and a large gastric residual volume, impairing the successful establishment of enteral feeding, which is critical for rapid growth and development in early life [1,2]. A systematic review reported a prevalence of approximately 27% for FI in preterm infants [3]. The reliance on parenteral nutrition in FI also leads to prolonged catheterization and an elevated risk of infection and cholestasis. The pathogenesis of FI remains largely unclear, and it is currently thought to be closely related to intestinal motility function, intestinal immunity, and the development of intestinal microbiome [1,2].

Concomitant with the maturation of the gastrointestinal system, the colonization and development of the intestinal microbiota occur rapidly in early life. The gut microbiota plays a crucial role in the development and maintenance of GI immunologic, sensory, and motor functions through direct or indirect interactions with the host [1]. Compared to full-term infants, hospitalized preterm infants face unique challenges due to prematurity and adverse environmental and host conditions before and after birth. As a result, their gut microbiota shows substantial inter-individual variation [4,5] and is often characterized by the reduction or absence of beneficial bacteria (e.g., *Bifidobacteria* and *Lactobacilli*) and enrichment of potentially pathogenic bacteria (e.g., *Klebsiella* and *Escherichia coli*) [6,7]. These microbial differences can be influenced by various demographic and environmental factors and pose impacts on the early development of the intestine [8]. Furthermore, the establishment of the microbiota in preterm infants is synchronized with and is closely related to neurological development, and dysbiosis-associated neurodevelopmental disorders may further affect their long-term prognosis [9,10].

Altered gut microbiome have been implicated in the progressions of FI. Several studies have explored the microbial alterations in preterm infants with FI, revealing reduced diversity, changes in microbial distribution, and an increased relative abundance of the *Proteobacteria* phylum and *Klebsiella* genus [11,12,13,14,15]. However, due to variations in study populations, sampling time points, and sampling frequency, definitive conclusions have not yet been reached.

The associations between gut microbiome and host GI development remain to be illustrated in the pathogenesis of FI. Calprotectin, a 36-kDa protein belonging to the S100 calcium-binding family, is abundant in neutrophils’ cytosolic proteins and is secreted at inflammation sites [16]. Fecal calprotectin (FC) has emerged as a reliable immune biomarker for assessing GI inflammation in adults and older children [17]. Recent studies have shed light on the critical role of calprotectin in the early postnatal development of the gut microbiota and the immune system, including the promotion of beneficial bacterial colonization and regulation of gut mucosal immunity, inflammatory processes, and cell development during infancy [18]. In our previous study, we observed significantly lower FC levels in infants with FI compared to non-FI infants during their NICU stay, suggesting a potential underdevelopment of the immune system in FI [19]. Therefore, investigating the associations between microbial alterations and changes in FC levels, along with exploring the underlying mechanisms, holds promise as a valuable approach to comprehending the role of the microbiome in shaping the intestinal microenvironment in FI infants.

In this study, we conducted a prospective study in a cohort of very preterm infants admitted to the NICU and consecutively collected stool samples, aiming to explore the postnatal dynamics and interplay between microbiota, metabolic profiles, and host immunity in preterm infants with and without FI. The findings provide novel insights to probe the complex interplay between the gut microbiome and host immunity in preterm infants and may contribute to the development of new strategies for the further regulation and promotion of GI health in this vulnerable population.

## 2. Materials and Methods

### 2.1. Study Design and Participants

We used a prospective cohort design and nested a case–control study within. The study was conducted in the NICU of Children’s Hospital of Fudan University in China from January 2018 to October 2020. In the prospective cohort, all infants with gestational age <32 weeks or with birth weight <1500 g and admitted to NICU within 24 h of life during the study period were enrolled and followed prospectively by the research team. The exclusion criteria include major congenital anomalies, inborn error of metabolism, sepsis, confirmed necrotizing enterocolitis (NEC), and hospitalization of fewer than seven days. Infants with fewer than two consecutive samples were further excluded from the study. The Ethics Committee of the Children’s Hospital of Fudan University approved this study (No. 2018(149), 20 June 2018). Oral informed consent was obtained from the parents of the participants.

### 2.2. Definitions

FI was defined when all the following criteria were fulfilled: (1) a gastric residual volume of >50% of the previous feeding volume; (2) emesis or abdominal distention or both; and (3) decreased, delayed, or discontinued enteral feeding [20]. NEC was defined as neonates with stage ≥ II NEC according to the Bell criteria [21]. Sepsis includes both culture-proven and culture-negative sepsis. Culture-proven sepsis was defined by a positive blood culture. Culture-negative sepsis was diagnosed when all the following criteria were fulfilled: (1) infection-related clinical manifestations; (2) abnormal white blood cell count, *C*-reactive protein level, or procalcitonin level; (3) antibiotics used or intended for ≥5 days; (4) negative blood culture; and (5) no evidence of concurrent focal infection [22,23,24,25]. Early-onset sepsis was defined as sepsis diagnosed within 72 h of postnatal age [26]. Gestational age was determined using the hierarchy of best obstetric estimates or gestational age estimation using the Ballard Score [27]. Small for gestational age was defined as birth weight <10th percentile for the gestational age according to the Chinese neonatal birth weight values [28]. Full enteral feeding was defined as receiving enteral feeding volumes of 120 mL per kilogram per day for 3 consecutive days [29].

### 2.3. Fecal Sample Collection, Preparation, and FC Measurement

Fecal samples were collected weekly from admission to discharge from all enrolled infants. Fecal samples were immediately stored at 0 °C and transferred to −80 °C within 1 h for long-term storage until thawed for further analysis. Meconium was defined as the stool passed within 72 h after birth.

FC levels were determined using a monoclonal ELISA kit (EK-CAL, Bühlmann, Schönenbuch, Switzerland), according to the manufacturer’s instructions. FC levels were expressed as micrograms per gram (µg/g) of feces. FC levels below the detection threshold (30 µg/g) were replaced with the minimum value (30 µg/g). Samples with FC values larger than the detection range (1600 µg/g) were further retested after dilution to obtain the exact values.

### 2.4. Stool 16S rRNA Gene Sequencing and Quantitative Microbial Profiling

The 16S rRNA sequencing and raw data preprocessing were processed according to the standard protocols provided by Majorbio Bio-Pharm Technology Co., Ltd. (Shanghai, China), as previously described [30]. In brief, microbial genomic DNA was isolated from each fecal specimen using the E.Z.N.A.^®^ Soil DNA Kit (Omega Bio-Tek, Norcross, GA, USA) according to the manufacturer’s instructions. The V3–V4 hypervariable region of the bacterial 16S rRNA gene was sequenced using universal primer pairs 338F (5′-ACTCCTACGGGAGGCAGCAG-3′) and 806R (5′-GGACTACHVGG GTWTCTAAT-3′). Chimera was detected and removed via UCHIME algorithm. Taxonomic identity was assigned to the resulting Operational Taxonomic Units (OTUs; 97% similarity) by alignment to the Silva (SSU123) 16S rRNA database using a confidence threshold of 70%. Samples with a total read of less than 20,000 were removed. Sequences that were present only once in each sample were further filtered.

Quantification of total bacteria was performed in triplicate, as previously described [31]. The absolute abundance of 16S rRNA genes relative to stool mass was measured using qPCR, targeting a conserved region of the gene with primers 338F and 806R. Standard curves were constructed using full-length 16S rRNA gene amplicons to convert threshold cycle (Ct) values to gene copy numbers per gram of stool. Taxon-specific copy numbers from the rrnDB database were used to correct the gene copy numbers [31,32].

Microbial composition and downstream analyses were carried out using the ‘phyloseq’ (ver. 1.30.0) package and “vegan” package (ver. 2.5-6) in R. Dominant bacterial taxa (phylum, family, or genus level) were defined as those with a prevalence of ≥20% in samples and an estimated abundance of ≥10^7^ cells per gram of stool. Between-sample diversity (beta diversity) was obtained by calculating unweighted UniFrac distances or Bray–Curtis distances at the taxonomic genus level and was visualized through principal coordinate analysis (PCoA). Permutational multivariate analysis of variance (PERMANOVA) was performed to determine factors that explained variance in bacterial community compositions of samples based on 2000 permutations.

### 2.5. Broad Range Metabolome by Liquid Chromatography–Mass Spectrometry

Liquid Chromatography–Mass Spectrometry (LC–MS) was performed using the ACQUITY UHPLC system (Waters Corporation, Milford, MA, USA) coupled with AB SCIEX Triple TOF 5600 System (AB SCIEX, Framingham, MA, USA). Metabolic profiling was analyzed in both ESI-positive and ESI-negative ion modes. The acquired raw data were processed using Progenesis QI (Waters Corporation) to obtain a two-dimensional data matrix containing retention time, positive mode, or negative mode mass-to-charge ratio [33], observations (sample), and peak intensity.

Statistical interrogations, data preprocessing, and integrated pathway analyses were conducted using the MetaboAnalyst 5.0 computational platform (https://metaboanalyst.ca, accessed on 13 October 2022) and ‘MetaboAnalystR’ [34] package (ver. 2.0.1) in R. Metabolites with RSD of >25% in QC pools were filtered out. For every metabolite feature, the missing values were replaced by a value half of the minimum peak intensity of the entire dataset. Quantile normalization and log transformation were applied to all metabolome data. All metabolites with annotated Human Metabolome Database (HMDB) IDs were included in downstream analysis [35]. Over-representation analysis was conducted in Metabolite Set Enrichment Analysis (MSEA) using the MetaboAnalyst 5.0 computational platform (https://metaboanalyst.ca, accessed on 21 November 2022). Enrichment scores represent the ratio of the Q-statistic for each pathway to the expected statistic of that pathway [36].

Fecal metabolite abundances were log-transformed and then weighted gene co-expression network analysis (WGCNA) was performed using the ‘WGCNA’ package [37]. Positively correlated metabolites were clustered together using ‘signed hybrid’ networks and biweight midcorrelation. Modules that correlated with each other at 0.85 or greater were merged. An eigen value was then applied to each module based on its position along the PC1 axis.

### 2.6. Statistical Analysis and Data Integration

With the exception of metabolic GSEA analyses, all statistical analyses and graphical outputs were performed using R (ver. 3.6.1). Graphs were created using the R packages ggplot2 and ggpubr. Parametric and non-parametric pairwise comparisons were made using the R package rstatix. Correlations between different indexes were explored using Spearman’s methods. Survival analyses were applied to determine the difference in time taken to reach full enteral feeding and hospitalization in infants with and without FI using the R package ‘survival’. Mixed-effects linear regressions were applied to determine the clinical associations of bacterial abundance or the correlation between FC level and bacterial abundance with infant ID as a random intercept. The continuous variables included in the models mentioned above were postnatal age (days), gestational age (weeks), birth weight (g), Apgar scores at 1 min, and days of antibiotic use or probiotic use within the past week (days). The binary variables include sex, delivery mode, multiple pregnancy, use of prenatal antibiotics, and exclusive breastfeeding. Metabolic modules and bacterial correlations were evaluated using the R package WGCNA. Procrustes analysis was performed on the profile of the fecal metabolome (Euclidian distances) and the 16S microbiota composition (unweighted UniFrac distances) using packages ape and vegan based on 1000 Monte Carlo iterations. Statistical significance for Procrustes was determined using ‘protest’. The false discovery rate (FDR) approach was used to control false positives in multiple testing. The bi-directional mediation analysis were applied to infer the causal role of the gut microbiome in contributing to the fecal calprotectin level through fecal metabolites using the mediate function from the R package mediation [38]. The significance level was determined by a *p*-value < 0.05 or an FDR *p*-value < 0.1.

## 3. Results

### 3.1. Infants, Maternal, and Sample Characteristics

**Figure 1:**
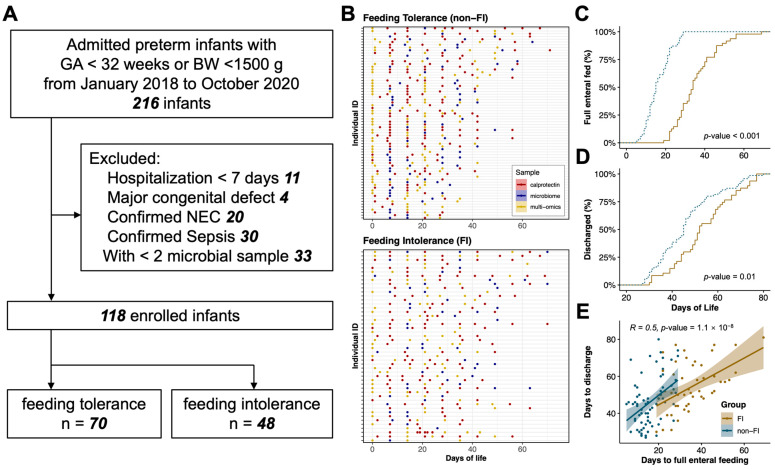
Study design, sample characteristics, and clinical differences in infants with and without feeding intolerance. (**A**) Flow chart of study design. (**B**) Fecal samples were collected at a weekly routine of all enrolled infants during hospitalization. X axis and Y axis represent different days of postnatal age and different infants, respectively. Fecal samples were all analyzed for calprotectin level (red color). Fecal samples in blue and yellow color were further analyzed for microbiome (blue color) or multi-omics (microbiome and metabolome, yellow color), respectively. (**C**,**D**) Differences in time to reach full enteral feeding (**C**) and days of hospitalizations (**D**) between infants with and without feeding intolerance, respectively. Yellow and blue color for infants with and without FI, respectively. (**E**) Correlations between days to full enteral feeding and days to discharge using Pearson’s method. Yellow and blue color for infants with and without FI, respectively.

From January 2018 to December 2020, a total of 118 very preterm infants without necrotizing enterocolitis or sepsis were included in the study (Figure 1A). We performed analyses on 674 fecal samples to determine FC levels, 348 samples for quantitative bacterial abundance (including 294 samples qualified for microbiome composition), and 225 samples for untargeted metabolome, respectively (Figure 1B).

The preterm infants were further categorized into two groups based on their feeding conditions during hospitalization: the feeding tolerance group (non-FI group), comprising 48 infants; and the feeding intolerance group (FI group), comprising 70 infants, respectively. Demographic variables between the two groups did not show significant differences (Table 1). However, infants in the FI group exhibited a prolonged duration to attain full enteral feeding (median (IQR): 34 (13) and 15 (9) days, respectively) and longer hospital stays (median (IQR): 54.4 (13.6) and 46.3 (13.3) days, respectively), and these two parameters were positively correlated with each other (Figure 1C–E).

### 3.2. Postnatal Dynamics of the Gut Microbiome and Metabolome in Very Preterm Infants

**Figure 2:**
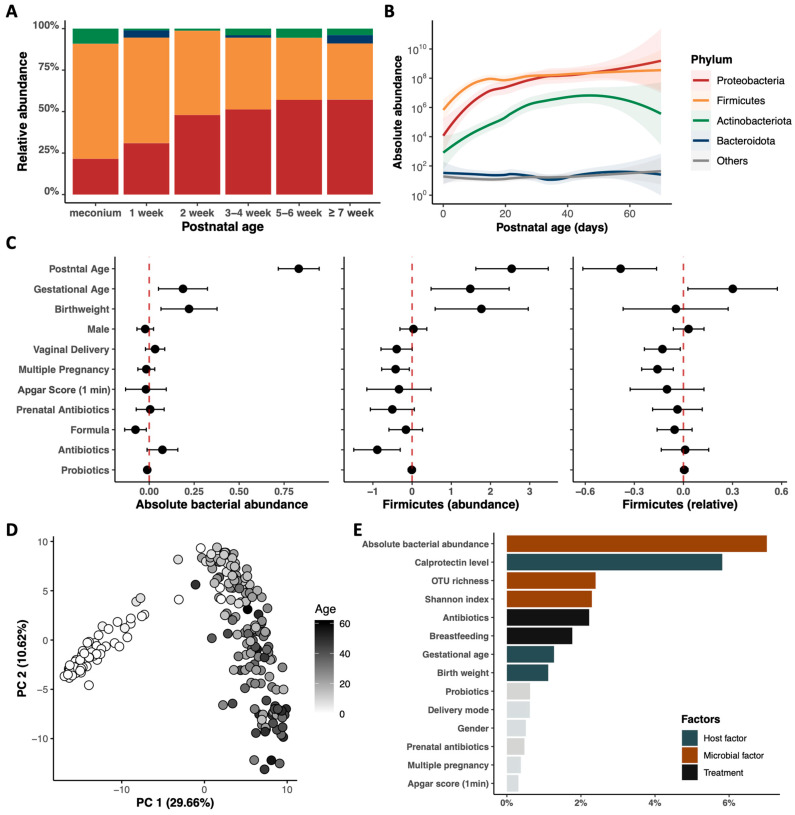
Postnatal dynamics and clinical associations of gut microbiome and metabolome in preterm infants in NICU. (**A**,**B**) Longitudinal postnatal development of gut microbiome at phylum level through compositional (**A**) and quantitative (**B**) perspectives, respectively (*n* = 294). (**C**) Effects of different clinical factors on absolute bacterial abundance, estimated absolute abundance of *Firmicutes*, and relative abundance of *Firmicutes*, respectively. Error bars indicate 95% confidence intervals. (**D**) Principal coordinate analysis (PCA) based on the Euclidean distance of the metabolic signatures of all samples. Dots in different grayscale images represent samples collected at different postnatal ages. (**E**) Multi-variate PERMANOVA analyses revealed the effects of different clinical factors on fecal metabolome profiles (*n* = 225). Percentages shown are the percentage of the variation explained by the corresponding factors, adjusted for postnatal age. Bars in gray color represent factors that did not have a significant impact on the variance of metabolome profiles (*p* > 0.05).

The developmental trajectory of the gut microbiome in very preterm infants during early life was investigated (Figure 2A,B). Firmicutes phylum dominated the intestinal flora of hospitalized preterm infants and was gradually replaced by *Proteobacteria* (Figure 2A and Figure S1A). Quantitative profiling indicated substantial variability in bacterial abundance among preterm infant samples, with total bacterial abundance and the estimated abundances of all dominant phyla significantly increasing with postnatal age, particularly during the first two weeks of life (Figure 2B and Figure S1B).

The impact of major demographic and clinical variables on gut microbiota was assessed, including the absolute bacterial abundance and the abundance of dominant phyla (Figure 2C and Figure S1C). Postnatal age had the most significant effect on the absolute abundance of bacteria and that of *Firmicutes* phylum, but interestingly, it had a negative effect on the relative abundance of the same bacteria (Figure 2C). Similarly, prenatal and postnatal antibiotic use significantly affected the estimated absolute abundance, but not the relative abundance of *Firmicutes* phylum.

The fecal metabolome profile showed a patterned progression with postnatal age in preterm infants (PERMANOVA: R^2^ = 17.5%, *p* < 0.001, Figure 2D), with bacterial abundance, FC levels, and alpha diversity (OTU richness and Shannon index) being the most important factors influencing changes in the fecal metabolome (PERMANOVA: R^2^ = 7.0%, *p* < 0.001, Figure 2E). Other clinical factors, including antibiotic use, breastfeeding, gestational age, and birth weight, also had a significant influence on metabolomic development.

### 3.3. Compromised Developmental Trajectory of the Gut Microbiome Is Associated with Altered Calprotectin Levels in Preterm Infants with Feeding Intolerance

**Figure 3:**
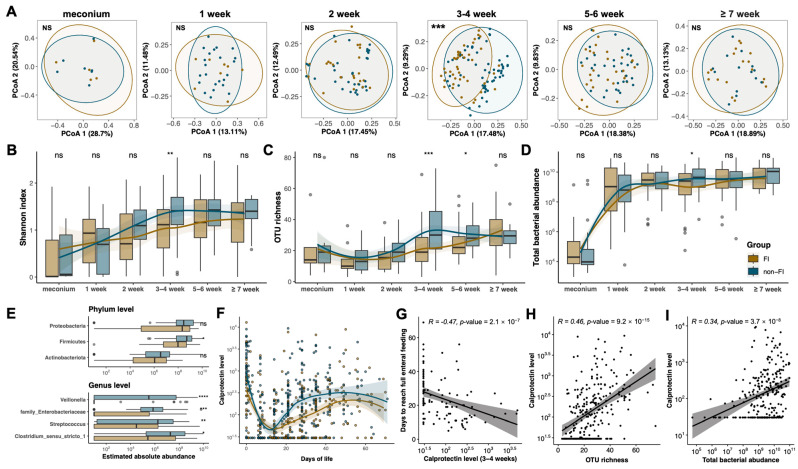
Compromised development of the gut microbiome is associated with altered calprotectin level in feeding intolerance. (**A**) Longitudinal change of microbiome profiles in infants with and without feeding intolerance (FI) using principal coordinate analysis (PCoA) based on the unweighted UniFrac dissimilarity at the genus level (*n* = 294). Percentages shown are the percentage of the variation explained by the corresponding principal coordinate, and the first two are displayed. (**B**–**D**) Longitudinal changes of Shannon index (**B**), OTU richness (**C**), and absolute bacterial abundance (**D**) in infants with and without FI, respectively. (**E**) The estimated bacterial abundance of all phyla and major genera of samples at 3–4 weeks of age that were significantly altered in infants with and without FI. (**F**) Longitudinal changes of fecal calprotectin (FC) levels in infants with or without FI, respectively. (**G**) Correlations between FC level at 3–4 weeks of age and time to reach full enteral feeding using Spearman’s method (*n* = 110). (**H**,**I**) Correlations between FC level and microbial OTU richness (**H**) and absolute abundance (**I**) in samples collected at and larger than 2 weeks of age using Spearman’s method (*n* = 235). Group-wise comparisons are shown using at-risk. Asterisks represent *p* values: ns: *p* > 0.05, * *p* < 0.05, ** *p* < 0.01, *** *p* < 0.001, **** *p* < 0.0001. Yellow and blue colors indicate infants with and without FI, respectively.

Next, we examined the developmental trajectory of the gut microbiome in infants with and without FI. The microbial composition in the FI group significantly differed from the non-FI group at 3–4 weeks of age (PERMANOVA: R^2^ = 4.8%, *p* < 0.001, Figure 3A). Additionally, during this period, the FI group exhibited reduced total bacterial abundance and alpha diversity within this period, as measured by the Shannon index and OTU richness (Figure 3B–D). Within the postnatal age of 3–4 weeks, differences in the estimated absolute abundance of dominant phyla and genera between the FI and non-FI groups were further analyzed (Figure 3E). At the phylum level, the estimated absolute abundance of the dominant phylum *Firmicutes* was significantly lower in the FI group compared to the non-FI group. At the genus level, significantly lower abundance of genera *Veillonella, Enterobacteriaceae* (*unknown genus*), *Streptococcus,* and *Clostridium_sensu_stricto_1* were observed in the FI group at 3–4 weeks of age (Figure 3E and Figure S1D).

Consistent with the altered microbiome, infants with FI had lower FC levels compared to those without FI, especially during 3–5 weeks of age (Figure 3F). Additionally, FC levels at 3–4 weeks of age were significantly negatively correlated with the time taken to reach full enteral feeding (Spearmen’s rho = −0.47, *p* = 2.1 × 10^−7^, Figure S2A) and the length of hospital stay (Spearmen’s rho = −0.4, *p* = 1.7 × 10^−5^, Figure 3G), respectively. From two weeks of age, the total bacterial abundance tended to stabilize after reaching a plateau, when FC levels started to show an increasing trend (Figure 3D,F). The associations between microbial features and FC levels were further explored. FC levels were significantly correlated with the OTU richness (Spearmen’s rho = 0.46, *p* = 9.2 × 10^−15^, Figure 3H), the total bacterial abundance (Spearmen’s rho = 0.34, *p* = 3.7 × 10^−8^, Figure 3I), and the inferred abundances of phyla *Firmicutes* and *Proteobacteria* and genus *Clostridium_sensu_stricto_1* in fecal samples collected at or older than 2 weeks of age (*p* < 0.05, Figure S2B–D).

### 3.4. Multi-Omics Analysis Identified Pantothenic Acid and Polyamine Metabolites as Potential Key Mediators in the Crosstalk between Gut Microbiota and Host Immunity

**Figure 4:**
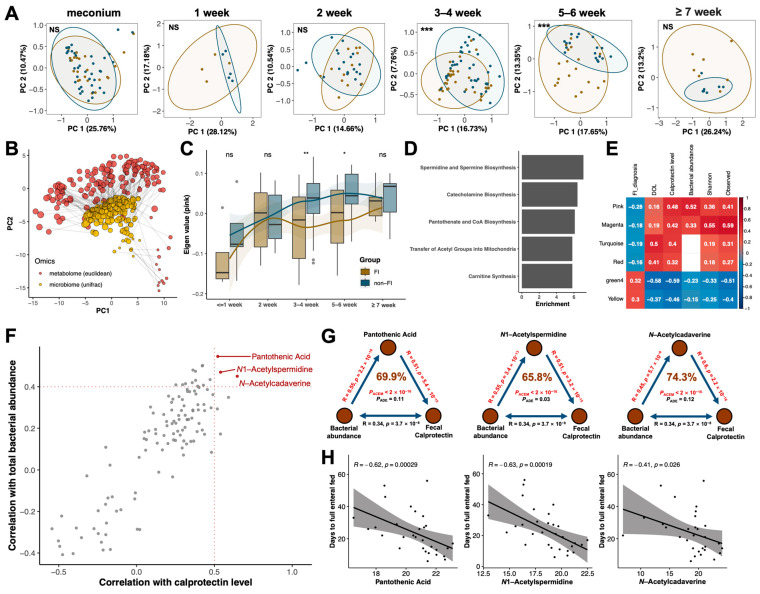
Potential roles of metabolites in the early development and crosstalk between microbiota and gut immunity. (**A**) Longitudinal change of metabolome profiles in infants with and without FI using principal components analysis (*n* = 225). Percentages shown are the percentage of the variation explained by the corresponding principal components, and the first two are displayed. (**B**) Correlation between the longitudinal development of microbiome and metabolome profiles using Procrustes analysis (*n* = 170). (**C**) Longitudinal change of eigen value of the ‘pink’ module in infants with and without feeding intolerance, respectively. (**D**) Top five enriched metabolic pathways from Small Molecule Pathway Database (SMPDB) in the ‘pink’ module using MSEA. The enrichment ratio represents the ratio of the Q-statistic for each pathway to the expected statistic. (**E**) Correlations between the eigen values of different metabolite modules and sample conditions (age and feeding volume at the time of collection), fecal calprotectin level, and microbial characteristics (total bacterial load and alpha diversity) using Spearman’s method. Only significant correlations are shown (*p* < 0.05). (**F**) Scatter plots reveal the correlations between intensities of metabolites in the ‘pink’ module and calprotectin level (X axis) and between metabolites intensities and total bacterial load (Y axis), respectively (*n* = 152). (**G**) Mediation linkages among the fecal bacterial abundance, metabolite intensity, and fecal calprotectin level. pACEM and pADE were estimated via the bidirectional mediation analysis. (**H**) Correlations between fecal metabolite intensities and days to reach full enteral feeding using Spearman’s method (*n* = 70). Group-wise comparisons are shown using at-risk. Asterisks represent *p* values: ns: *p* > 0.05, * *p* < 0.05, ** *p* < 0.01, *** *p* < 0.001. Yellow and blue colors indicate infants with and without feeding intolerance, respectively.

We then focused on the metabolic change and its roles in the microbiota–host interactions in very preterm infants. Longitudinal analysis of fecal metabolome profiles in very preterm infants revealed distinct differences between the FI group and non-FI group at 3–4 weeks (PERMANOVA: R^2^ = 4.2%, *p* < 0.001) and 5–6 weeks of postnatal age (PERMANOVA: R^2^ = 8.4%, *p* < 0.001) (Figure 4A). Procrustes analysis demonstrated a strong correlation between the metabolic niche and microbiota profiles during early life (R^2^ = 55.3%, *p* = 0.001, Figure 4B). To identify specific metabolic shifts associated with FI, all 1908 detected metabolites with HMDB annotations were clustered into 12 highly correlated modules using the WGCNA analysis (Figure S3A). Among the modules significantly associated with FI (Figure S3B), the eigen value of the ‘pink’ module, which contains 54 metabolites, exhibited the most significant alterations in the FI group compared to the non-FI group, particularly during 3–6 weeks of age (Figure 4C). MSEA revealed that major metabolic pathways in the ‘pink’ module included Spermidine and Spermine Biosynthesis, Catecholamine Biosynthesis, and Pantothenate and CoA Biosynthesis (Figure 4D). Regarding multi-omics correlations, the eigen value of the ‘pink’ module showed a weak association with postnatal age (Spearmen’s rho = 0.16) but exhibited the strongest correlations with FC levels (Spearmen’s rho = 0.48) and bacterial abundance (Spearmen’s rho = 0.52), suggesting its potential involvement in microbiota–host interactions (Figure 4E). Furthermore, correlations between the intensity of all metabolites in the ‘pink’ module and FC levels and bacterial abundance were examined in samples collected at or after two weeks of age (Figure 4F). Notably, pantothenic acid and two polyamines (*N*1-Acetylspermidine and *N*-Acetylcadaverine) showed the strongest associations with FC levels and total bacterial abundance (Figure 4F and Figure S3C). Mediation analyses revealed that these metabolites had high mediating effects, explaining 69.9%, 65.8%, and 74.3% of the effects of bacterial abundance on FC, respectively (Figure 4G). Moreover, the levels of these metabolites at three to four weeks of age were negatively correlated with the time taken to achieve full enteral feeding (Figure 4H) and the length of hospital stay (Figure S3D), respectively.

## 4. Discussion

In this longitudinal case–control study, we examined the developmental trajectory of the gut microbiome, metabolome, and gut immunity marker in preterm infants with and without feeding intolerance. Coordinate alterations in microbial and metabolic profiles were observed in FI within a critical time window. Notably, we identified a robust correlation between bacterial abundance and calprotectin level during early life, and polyamine metabolites and pantothenic acid were identified as potential key mediators in the imbalanced microbiome–immunity crosstalk observed in FI infants. These findings provide novel insights to probe the complex interplay between the gut microbiome and host immunity in preterm infants and may offer novel insights into underlying pathogenesis and potential regulation of GI health in preterm infants.

Feeding intolerance is a common clinical phenomenon in hospitalized preterm infants, with no widely accepted diagnostic criteria, leading to subjective and inconsistent diagnoses among practitioners and centers [3]. In this cohort, despite no significant differences in demographic characteristics between the non-FI and FI groups, FI had a significant impact on the time required to achieve full enteral feeding and a prolonged hospitalization duration. These results underscored the clinical importance of addressing FI in preterm infants to optimize their feeding progress and improve overall patient outcomes.

The early colonization of gut microbiome may be associated with GI development in preterm infants and contribute to the pathogenesis of FI. However, studies characterizing the gut microbiome development in FI infants are limited, with inconsistent results, likely due to variations in sampling points and populations. For instance, Yuan et al. [15] reported an increase in the relative abundance of *Klebsiella* and Liu et al. [12] reported a decrease in the relative abundance of *Clostridia* in FI infants, whereas Liu et al. [11] found no significant differences in the main microbial composition in infants with or without FI.

Through quantitative microbial profiling, we observed that the critical period of rapid microbial colonization occurred within two weeks after birth. It is also noteworthy that absolute quantification and relative abundance analysis may yield inconsistent or even contradictory information when examining trends of specific bacteria and associated influencing factors, given the significant increase in bacterial abundance during this period. Thus, the insights gained from absolute quantification deserve more attention and could provide valuable insights into the gut microbiome of preterm infants. We observed that changes in the microbiome of FI infants were characterized by significantly lower alpha diversity and bacterial abundance at 3–4 weeks of age, coinciding with the time period when the clinical manifestations of FI often occur. The bacterial characteristics may suggest a developmental lag in the microbiota during this period and a possible contribution to the impaired intestinal function observed in infants with FI.

As recent studies have highlighted the critical role of calprotectin in the early postnatal development of gut microbiota and the intestine of infants [18], the combined analysis of FC level and microbiome may help to explore the change of intestinal microenvironment in preterm infants with FI. Interestingly, we observed a strong association between postnatal FC level and the time to reach full enteral feeding, which suggested a potential role of FC level in overall GI development in this specific population [19]. Interestingly, a sharp rebound in FC levels was observed shortly after the rapid growth of intestinal flora, and the robust correlations between FC levels and microbial abundance and diversity may, in part, reflect the interaction and synergetic development of gut microbiome and host immunity during the early postnatal age. Meanwhile, the simultaneous alterations in the microbiome and FC levels in infants with FI during the time frame of 3–4 weeks of age may suggest a disturbance of microbial–host interactions in the pathogenesis of FI.

The patterned and coordinated development of the gut microbiome and metabolome in early life emphasizes the key role of metabolites as mediators in microbiota–host interactions. By clustering and correlating metabolites with intestinal microbiome and calprotectin, we screened for three metabolites, pantothenic acid and two polyamines, that were significantly reduced in infants with FI and strongly correlated with FC levels and bacterial abundance. Remarkably, these metabolites exhibited high mediating effects on the effect of bacterial abundance on FC levels.

Decreased levels of polyamines in infants with FI may be associated with gut dysbiosis, as suggested by their strong association with bacterial abundance. Intestinal polyamines are derived from both the host and intestinal flora [39,40,41]. Dietary polyamines are absorbed in the upper gastrointestinal tract; thus, the major source of fecal polyamines is likely derived from the intestinal microbiota [41,42,43]. The mediating role of polyamine levels in the effect of microbiome on calprotectin levels may be closely linked to their impact on postnatal intestinal development and maintenance, especially the promotion of gut immunity. As previously reported, polyamines play a vital role in enhancing gut epithelial renewal by regulating proliferation, growth arrest, and apoptosis [41,44,45,46,47,48,49,50,51,52,53], modulating gut epithelial barrier function by influencing intercellular junctions and epithelial defense [39,54], and affecting the maturation of the intestinal immune system and the differentiation of intraepithelial lymphocytes and macrophages [41,49,55,56].

Similarly, pantothenic acid’s effects on intestinal function have been reported, with a deficiency of its transport protein being associated with growth retardation, spontaneous and severe inflammation, abnormal histology in the intestine, and altered gut permeability [57]. The close association between pantothenic acid and the intestinal flora is demonstrated by the fact that a de novo synthesis of pantothenic acid was restricted to the genomes of *Bacteroidetes* and *Proteobacteria*, whereas specific bacteria such as *Lactobacillus* spp., *Streptococcus* spp., and *Enterococcus* spp. require pantothenic acid for growth in vitro [58,59].

The close association of polyamines and pantothenic acid with flora and their multifaceted effects on gut health may largely explain their important mediating role in flora–host interactions, which requires further mechanistic studies. The discovery of these intermediates of the gut microbiome regulation of immune development may provide potential therapeutic targets for intervening in dysbiosis-associated gut dysfunction and improving prognosis in feeding intolerant infants.

Our study’s strengths include a relatively large sample size, longitudinal sampling throughout hospitalization, and in-depth analyses with detailed clinical information. However, there are several limitations. First, despite the fact that we evaluated the detailed clinical information of all patients and excluded confirmed NEC and sepsis in this study, the clinical definition of feeding intolerance may still be vague and could not be clearly distinguished from that of milk allergy and stage I NEC at an early stage. Therefore, population heterogeneity in infants with feeding intolerance may exist. Second, we only included very preterm infants with gestational age < 32 weeks or birth weight < 1500 g. The results need to be validated via targeted metabolomic analysis using a larger cohort. Lastly, the underlying mechanisms of microbiome–host interactions and the possible mediating roles of polyamine metabolites and pantothenic acid should be explored and validated through more in-depth experiments.

## 5. Conclusions

Our study revealed a compromised developmental trajectory of the microbiome–metabolome–immune axis in FI infants. We highlighted a robust correlation between bacterial abundance and calprotectin level during early life, and polyamine metabolites and pantothenic acid were identified as potential key mediators in the imbalanced microbiome–immunity crosstalk in FI infants. These findings provide novel insights to probe the complex interplay between the gut microbiome and host immunity and may potentially devise new strategies for the further regulation and promotion of GI health in this vulnerable population.

## Figures and Tables

**Table 1:** Demographics and clinical variables of infants with and without FI.

Variable	FI (*n* = 48)	Non-FI (*n* = 70)	*p* Value
Infant characteristics			
Gestational age (weeks), mean (SD)	29.8 (1.5)	30.2 (1.7)	0.208
Birth weight (g), mean (SD)	1265.2 (177.2)	1335.1 (202.3)	0.055
Male, *n*/*N* (%)	25 (52.1)	29 (41.4)	0.341
Small for gestational age, *n*/*N* (%)	3 (6.2)	6 (8.6)	0.909
Apgar score at 1 min, mean (SD)	7.6 (1.8)	7.9 (1.6)	0.318
Maternal characteristics			
Multiple pregnancy, *n*/*N* (%)	22 (45.8)	28 (40.0)	0.660
Vaginal delivery, *n*/*N* (%)	16 (33.3)	23 (32.9)	1.000
PPROM ^a^, *n*/*N* (%)	11 (22.9)	17 (24.3)	1.000
Use of prenatal antibiotics, *n*/*N* (%)	11 (22.9)	10 (14.3)	0.337
Gestational diabetes mellitus, *n*/*N* (%)	12 (25.0)	13 (18.6)	0.542
Gestational hypertension, *n*/*N* (%)	6 (12.5)	7 (10.0)	0.899
Treatment			
Early antibiotics use ^b^ (days), mean (SD)	4.2 (2.2)	3.6 (2.2)	0.128
Exclusive breastmilk feeding, *n*/*N* (%)	40 (81.4)	57 (91.6)	0.656
Probiotics use ^c^ (days), mean (SD)	6.2 (12.0)	5.3 (8.4)	0.656
Drugs for PDA ^d^, *n*/*N* (%)	8 (16.7)	6 (8.6)	0.296
Proton pump inhibitors, *n*/*N* (%)	1 (2.1)	2 (2.9)	1.000

^a^ PPROM, prolonged premature rupture of membrane; ^b^ Early antibiotics use in both groups were calculated as the accumulated days of antibiotics use within first week of age; ^c^ Probiotics use in both groups were calculated as the accumulated days of probiotics use throughout hospitalization; ^d^ Drugs for PDA include ibuprofen and acetaminophen.

## Data Availability

The 16S rRNA sequencing files were submitted to the National Center for Biotechnology Information Sequence Read Archive (https://www.ncbi.nlm.nih.gov/sra, accessed on 1 October 2023) and are available with BioProject accession number PRJNA996610. The fecal metabolite data have been made available on Figshare.com via the link: https://figshare.com/account/articles/23702871 (accessed on 1 October 2023).

## Supplementary Materials

The following supporting information can be downloaded at: https://www.mdpi.com/article/10.3390/nu15224849/s1.
