# Supplementary material for: Dynamics and Crosstalk between Gut Microbiota, Metabolome, and Fecal Calprotectin in Very Preterm Infants: Insights into Feeding Intolerance
Source: Nutrients. 2023 Nov 20;15(22):4849. doi: 10.3390/nu15224849 (PMC10674500; doi:10.3390/nu15224849)
Supplement: Supplementary file 1 [file nutrients-15-04849-s001.zip › nutrients-2698891-supplementary.pdf]

Supplementary materials

Dynamics and Crosstalk between Gut Microbiota, Metabolome, and Fecal Calprotectin in Very Preterm Infants: Insights into Feeding Intolerance

**Figure S1**	Longitudinal change of microbiome composition and estimated bacterial abundance in very preterm infants with and without feeding intolerance.	Page 2-3
**Figure S2**	Correlations between the fecal calrpteoctin level and FC levels.	Page 4
**Figure S3**	Clustering of metabolome profiles and correlations between bacterial load and intensity of polyamine metabolites.	Page 5

Fig S1. Longitudinal change of microbiome composition and estimated bacterial abundance in very preterm infants with and without feeding intolerance

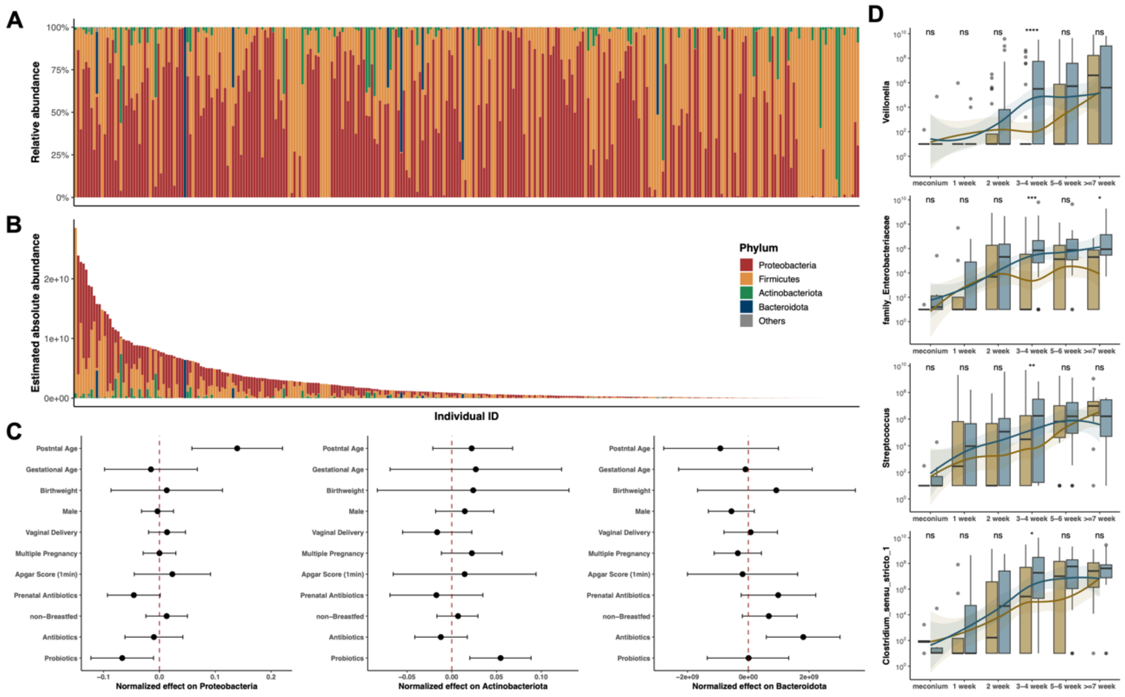

(A-B) Longitudinal change of bacterial composition **(A)** and estimated absolute abundance **(B)** in fecal samples of very preterm infants. **(C)** Effects of different clinical factors on the estimated absolute abundance of *Proteobacteria*, *Firmicutes*, *Actinobacteria*, and *Bacteroidota*. Error bars indicate 95% confidence intervals. **(D)** Longitudinal change of inferred bacterial abundance of genera *Veillonella, Enterobacteriaceae, Streptococcus, and Clostridium_sensu_stricto_1* in infants with and without FI, respectively. Yellow and blue color for infants with and without FI, respectively. Group-wise

comparisons were shown using at-risk. Asterisks represent p values: ns: $p > 0.05$, *$p < 0.05$, **$p < 0.01$, ***$p < 0.001$, ****$p < 0.0001$.

Fig S2. Correlations between the fecal calrpteoctin level and FC levels

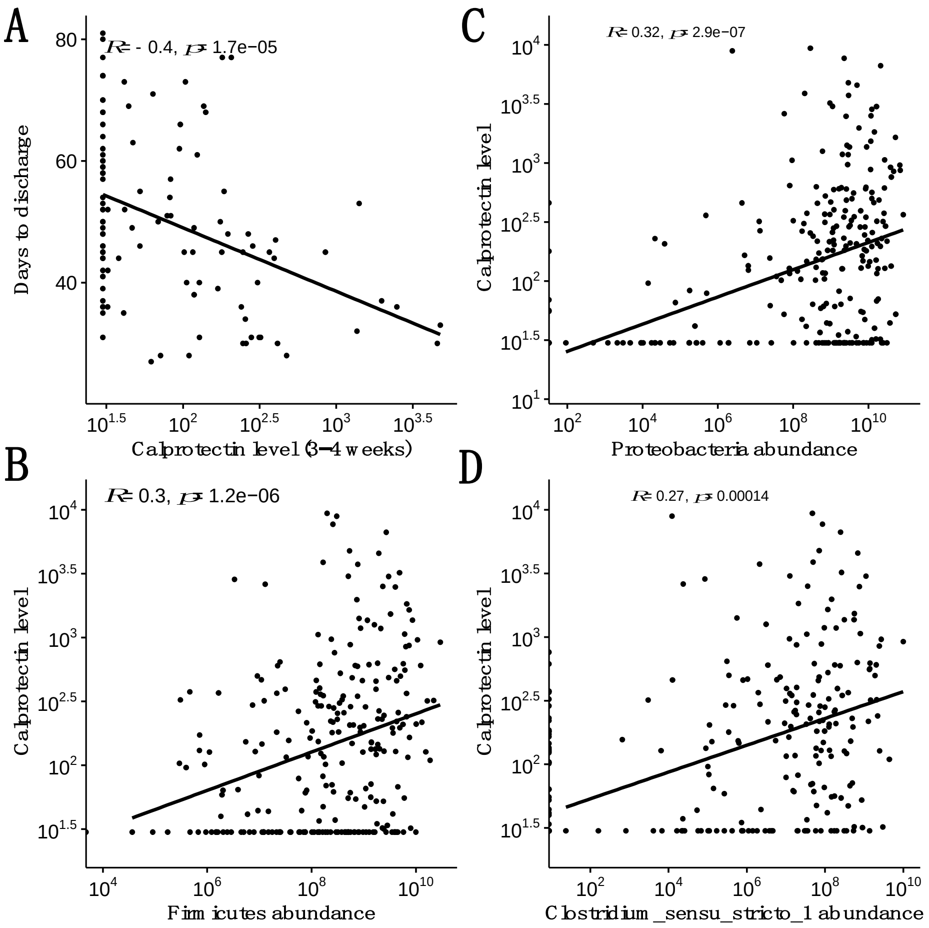

(A) Correlations between fecal calprotectin level at 4 weeks of age and time to discharge using Spearman’s method. **(B-D)** Correlations between inferred bacterial abundances of phyla *Firmicutes* **(B)** and *Proteobacteria* **(C)** and *genus Clostridium_sensu_stricto_1* **(D)** in samples collected at and large than 2 weeks of age using Spearman’s method, respectively.

Fig S3. WGCNA analyses of metabolome profiles and Spearman's correlations between total bacterial load and intensity of polyamine metabolites

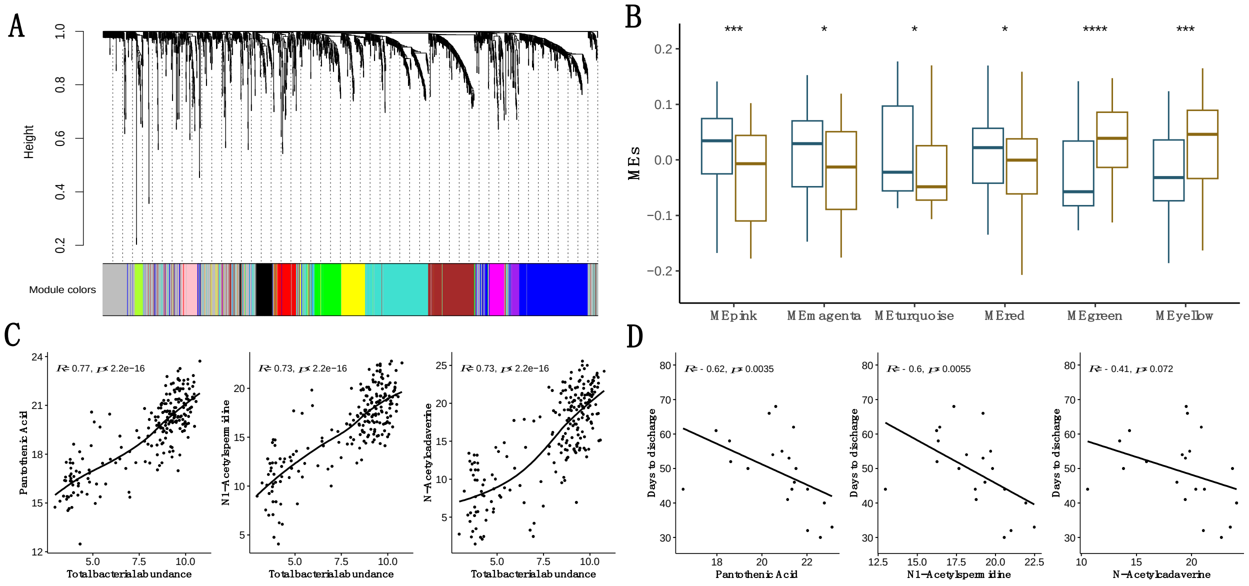

(A) WGCNA clustering of all metabolites with HMDB annotations. **(B)** The overall changes of different metabolite modules in infants with and without FI, respectively. Group-wise comparisons were shown using at-risk. Asterisks represent p values: ns: $p > 0.05$, *$p < 0.05$, **$p < 0.01$, ***$p < 0.001$, ****$p < 0.0001$. Yellow and blue color for infants with and without FI, respectively. **(C)** Correlations between fecal metabolite intensities and total bacterial abundance using Spearman's method in infants with and without FI, respectively. **(D)** Correlations between fecal metabolite intensities and days to discharge using Spearman's method in infants with and without FI, respectively.
